# Frequency-Dependent Brain Regional Homogeneity Alterations in Patients with Mild Cognitive Impairment during Working Memory State Relative to Resting State

**DOI:** 10.3389/fnagi.2016.00060

**Published:** 2016-03-24

**Authors:** Pengyun Wang, Rui Li, Jing Yu, Zirui Huang, Juan Li

**Affiliations:** ^1^Key Laboratory of Mental Health, Center on Aging Psychology, Institute of Psychology, Chinese Academy of SciencesBeijing, China; ^2^Faculty of Psychology, Southwest UniversityChongqing, China; ^3^Institute of Mental Health Research, University of OttawaOttawa, ON, Canada

**Keywords:** mild cognitive impairment, working memory, state, regional homogeneity, frequency bands

## Abstract

Several studies have reported working memory deficits in patients with mild cognitive impairment (MCI). However, previous studies investigating the neural mechanisms of MCI have primarily focused on brain activity alterations during working memory tasks. No study to date has compared brain network alterations in the working memory state between MCI patients and normal control (NC) subjects. Therefore, using the index of regional homogeneity (ReHo), we explored brain network impairments in MCI patients during a working memory task relative to the resting state, and identified frequency-dependent effects in separate frequency bands.Our results indicate that, in MCI patients, ReHo is altered in the posterior cingulate cortex (PCC) in the slow-3 band (0.073–0.198 Hz), and in the bottom of the right occipital lobe and part of the right cerebellum, the right thalamus, a diffusing region in the bilateral prefrontal cortex (PFC), the left and right parietal-occipital regions, and the right angular gyrus in the slow-5 band (0.01–0.027 Hz). Furthermore, in NCs, the value of ReHo in clusters belonging to the default mode network (DMN) decreased, while the value of ReHo in clusters belonging to the attentional network increased during the task state. However, this pattern was reversed in MCI patients, and was associated with decreased working memory performance. In addition, we identified altered functional connectivity of the abovementioned regions with other parts of the brain in MCI patients. This is the first study to compare frequency-dependent alterations of ReHo in MCI patients between resting and working memory states. The results provide a new perspective regarding the neural mechanisms of working memory deficits in MCI patients, and extend our knowledge of altered brain patterns in resting and task-evoked states.

## Introduction

Mild cognitive impairment (MCI) is a syndrome in which individuals display certain forms of cognitive dysfunction but are still able perform basic daily activities. MCI is generally considered to be a transitional stage between normal aging and clinical dementia (Petersen, 2004). A meta-analysis of MCI patients reported that the annual conversion rate from MCI to dementia was approximately 5–10% (Mitchell and Shiri-Feshki, 2009), which is significantly higher than the incidence of dementia in normal elderly patients (1–2% per year; Petersen, 2004).

Many studies have evaluated working memory deficits in MCI patients (Klekociuk and Summers, 2014; Kirova et al., 2015). Rapid declines in working memory as well as delayed recall and spatial memory have been reported following the conversion of MCI to Alzheimer’s disease (AD; Cloutier et al., 2015). Studies investigating the corresponding neural mechanisms of MCI have examined altered brain activation during working memory tasks (Bokde et al., 2010; Lou et al., 2015; Migo et al., 2015); however, the pattern of intrinsic brain oscillations during a working memory task has not yet been explored for comparison. Studies have also evaluated functional network differences between resting and task states. For example, it has been found that functional connectivity (FC) is altered in specific networks during visual stimulation (Nir et al., 2006), during a motor task (Jiang et al., 2004), and during sustained attention (He, 2011; You et al., 2013). Furthermore, alterations between resting and task states have been associated with task performance in children with autism spectrum disorders (You et al., 2013). Of note, Lou et al. (2015) evaluated background network efficiency (an index based on graph theory) during a working memory task and reported increased background network efficiency in MCI patients that was hypothesized to compensate for decreased working memory capacity (Lou et al., 2015). However, no study to date has evaluated the impairment of specific brain network regions or the properties of associated brain networks (e.g., functional connectivity [FC], regional homogeneity [ReHo], and amplitude of low-frequency fluctuations [ALFF]) in MCI patients in resting vs. working memory states. Additionally, the relationship between network abnormalities and working memory deficits in MCI is still unknown.

The index of ReHo employs Kendall’s coefficient of concordance (KCC) to measure the coordination of activity between voxels within a region, and thus reflects intra-regional synchronization (Zang et al., 2004). ReHo can be considered as a complementary method to model-driven methods, and can be utilized to investigate the complexities of human brain function (Zang et al., 2004). As an index of intrinsic brain activation, ReHo has been successfully used to reflect differences between resting and active brain states (Zang et al., 2004; Huang et al., 2014). In the present study, we used this index to investigate altered brain oscillations during the resting vs. working memory states in MCI patients.

Furthermore, we studied blood oxygenation level-dependent (BOLD) frequency-dependent effects (Buzsáki and Draguhn, 2004; Hoptman et al., 2010; Zuo et al., 2010; Han et al., 2011; Huang et al., 2014) of ReHo in separate bands, as frequency-dependent effects in different brain regions have been hypothesized to reflect synaptic/functional/cytoarchitectonic characteristics (He et al., 2010; Baria et al., 2011) that are affected by the progression of cognitive impairment. Using the ALFF index, Han et al. (2011) found that MCI patients demonstrate altered patterns in specific frequency bands as compared to normal control (NC) elderly individuals in the resting state (Han et al., 2011). However, frequency-dependent changes of ReHo in MCI patients and effect interactions during the resting and working memory states are still unknown. In the present study, we divided the entire frequency range of the BOLD fluctuation signal (0–0.25 Hz) into five bands: slow-6 (0–0.01 Hz), slow-5 (0.01–0.027 Hz), slow-4 (0.027–0.073 Hz), slow-3 (0.073–0.198 Hz), and slow-2 (0.198–0.25 Hz; Buzsáki and Draguhn, 2004; Hoptman et al., 2010; Zuo et al., 2010). We mainly focused on the slow-5, slow-4, slow-3, and slow-2 bands, as the signal of slow-6 was considered to primarily reflect very low frequency drift (Biswal et al., 1995; Zuo et al., 2010). Due to large signal fluctuations during tasks, we expanded the maximum threshold of BOLD from the traditional 0.1 to 0.25 Hz, which is the highest frequency of data that can be sampled when time repetition (TR) = 2000 ms (Buzsáki and Draguhn, 2004; Zuo et al., 2010).

In sum, the goal of the present study was to examine whether changes in ReHo during resting and working memory states differ between MCI patients and NC subjects. The present study also aimed to identify a correlation between behavioral performance in a working memory task and patterns of altered activity in MCI patients. Finally, we explored the distant connectivity of regions showing a significant interaction of group and state for ReHo in MCI patients vs. NC subjects.

## Materials and Methods

### Participants

In total, 17 MCI patients and 16 healthy NC elderly adult subjects participated in this study. Participants were recruited from a community-based screening data pool in Beijing (healthy older adults, *n* = 865; MCI, *n* = 115; dementia, *n* = 21; Yu et al., 2012, 2014; Yin et al., 2015). Each participant completed a series of neuropsychological tests, clinical assessments, and neuroimaging examinations and was diagnosed by an experienced psychiatrist. MCI was diagnosed according to the diagnostic criteria for MCI (Petersen et al., 1999, 2001) and supplemented by scores from the Montreal Cognitive Assessment (MoCA; Nasreddine et al., 2005), Mini-Mental Status Examination (MMSE; Folstein et al., 1975), and Clinical Dementia Rating (CDR; Morris, 1993). A summary of participant demographics and neuropsychological test results is reported in Table 1. This study was approved by the research ethics committees of the Institute of Psychology, Chinese Academy of Science (H11036). Written informed consent was obtained from each participant.

**Table 1:** **Summary of participant demographic information**.

	NC	MCI	*p* value
*n*	16	17	—
Sex (male/female)	8/8	9/8	0.866
Age (years)	68.56 ± 5.76	70.53 ± 4.54	0.283
Education (years)	11.75 ± 3.17	9.82 ± 4.63	0.176
Self-rating anxiety scale	25.25 ± 4.51	29.08 ± 6.13	0.063
ADL	14.19 ± 0.54	15.15 ± 2.30	0.115
MMSE	28.25 ± 1.39	24.47 ± 3.88	<0.010
MoCA	26.19 ± 1.52	19.18 ± 4.45	<0.001
CDR	0	0.5	—
Accuracy	91.12 ± 4.64%	77.50 ± 17.98%	0.007
Response time	609.76 ± 56.09	692.86 ± 86.36	0.003
Working memory performance	1.51 ± 0.15 (×10^#x02212;3^)	1.16 ± 0.36 (×10^#x02212;3^)	0.001

*Abbreviations: NC, normal control; MCI, mild cognitive impairment; ADL, Activities of Daily Life; MMSE, the Mini-Mental Status Examination; MoCA, the Montreal Cognitive Assessment; CDR, the Clinical Dementia Rating*.

### The Hybrid Delayed-Match-to-Sample Task (DMST)

To evaluate working memory, participants were instructed to memorize 32 pictures by performing a naming task prior to scanning. Participants then completed a memory test. After initial testing, participants performed a working memory task in an fMRI scanner. The working memory task, which was modified from a previously reported delayed match-to-sample paradigm, consisted of 32 trials (30 s each) separated into four blocks (8 trials per block; Jiang et al., 2000; Lawson et al., 2007; Guo et al., 2008). During each trial, two target objects with green borders were presented side-by-side for 3500 ms. Participants were instructed to memorize the target objects. Targets were then followed by test objects presented for 1000 ms each with variant jitters at 800/900/1000/1100/1200 ms. Test objects were matching target objects or non-matching distractor objects. Target objects and distractor objects were presented 2–4 times each for a total of 12–13 test objects per trial. The presentation of target and distractor objects in each trial was pseudo-randomized and counter-balanced. Participants were asked to indicate when a test object matched a target object by pressing a button with their right thumb, and when a test object did not match one of the target objects by pressing another button with their left thumb. Button assignments were counterbalanced among participants. Of note, half of the targets and half of the distractors had already been viewed and studied by participants prior to scanning, but the significance of this was not evaluated. To control for the effects of visual processing, responses to scrambled versions of object images containing the same spatial frequencies were used as a baseline. Participants were asked to press both buttons in response to scrambled pictures. Each scrambled picture block contained five scrambled pictures presented for 2000 ms each. Scrambled picture blocks were alternated between DMST memory trials.

### Image Acquisition

Participants were scanned using a Siemens Trio 3.0 tesla scanner (Erlangen, Germany) at the Beijing MRI Center for Brain Research. During resting state scanning, participants were instructed to lie quietly with their eyes closed and not to think of anything in particular. For each participant, 200 resting state functional volumes were collected using the following parameters: TR = 2000 ms, flip angle = 90°, time echo (TE) = 30 ms, thickness = 3.0 mm, field of view (FOV) = 200 × 200 mm^2^, 33 axial slices, acquisition matrix = 64 × 64, gap = 0.6 mm, and in-plane resolution = 3.125 × 3.125. During working memory task scanning, the same parameters were used to collect 163 functional volumes for each run. Additionally, high-resolution 3-dimensional T1-weighted structural images were obtained for each participant using the following parameters: TR = 1900 ms, TE = 2.2 ms, flip angle = 9°, acquisition matrix = 256 × 256, 176 slices, and voxel size = 1 × 1 × 1 mm^3^.

### Behavioral Data Analysis

The response accuracy of total working memory was calculated according to the total hit rate (correct target detection) minus the total false alarm rate (false report for distractors). Response times (RTs) were calculated as the mean RT for all test stimuli (targets and distractors). To consider the tradeoffs between accuracy and RT, working memory performance was further indexed using response accuracy divided by RT, which is the reciprocal of the “inverse efficiency score” previously reported (Kennett et al., 2001; Spence et al., 2001).

### Image Preprocessing

Functional MRI data, including both resting and task states, were preprocessed using the Statistical Parametric Mapping program (SPM8^1^) and the toolbox for Data Processing and Analysis of Brain Imaging (DPABI V1.3^2^; Yan and Zang, 2010). The first nine volumes of working memory task data were discarded to allow for equilibration of the magnetic field. To acquire equal volumes between resting and working memory states, the first 46 volumes of resting state data were discarded. The remaining 154 volumes of both states were corrected for intra-volume acquisition time differences between slices using Sinc interpolation. Then, volumes were corrected for inter-volume geometrical displacement due to head motion using a 6-parameter spatial transformation. All included participants had head motions less than 3 mm in any one direction during scanning. Coregistration, segmentation, and writing normalization were conducted using unified segmentation of each participant’s T1 image. Normalized volumes were resampled to a voxel size of 3 × 3 × 3 mm^3^. Nuisance covariates, including head motion parameters, global mean signal, white matter signal, and cerebral spinal fluid signal, were regressed out. fMRI images were further spatially normalized to the Montreal Neurological Institute (MNI) echo planar imaging (EPI) template using an optimized12-parameter affine transformation and nonlinear deformations. Finally, temporal band-pass filtering was performed at slow 5 (0.01–0.027 Hz), slow 4 (0.027–0.073 Hz), slow 3 (0.073–0.198 Hz), and slow 2 (0.198–0.25 Hz) frequencies, respectively. As spatial smoothing may have artificially enhanced ReHo intensity and reduced its reliability (Zuo et al., 2013), ReHo was calculated from an unsmoothed BOLD time series. Spatial smoothing was then performed using a 4-mm full-width at half-maximum (FWHM) Gaussian kernel.

### Whole Brain Regional Homogeneity (ReHo)

ReHo analysis was performed for each subject using the DPABI program mentioned above. Specifically, for each voxel in the whole brain, KCC was calculated between the voxel’s BOLD time series and those of its 26 nearest neighboring voxels (Zang et al., 2004) to yield a voxel-wise ReHo map. All individual ReHo maps were computed and standardized into ReHo *Z*-values by subtracting the mean voxel-wise ReHo obtained for the entire brain (i.e., global ReHo), and then dividing the resultant value by the standard deviation (Zuo et al., 2013). This subject-wise ReHo normalization has been demonstrated to improve normality and reliability across subjects (Zuo et al., 2013).

### Structural Image Analysis

Previous studies have reported gray matter (GM) atrophy in regions such as the medial temporal lobe, the frontal cortex, and the parietal cortex of MCI patients (Singh et al., 2006; Karas et al., 2008), To control for the influence of GM atrophy on our ReHo analysis, a voxel-based morphometry (VBM) analysis was performed for structural images using DPABI to identify regions of GM atrophy. Structural images of each participant were coregistered to mean functional images after motion correction using a linear transformation, and then segmented into GM, white matter, and cerebrospinal fluid in the MNI space (Ashburner and Friston, 2005). Then, we performed a voxel-based 2-sample *t*-test on GM intensity maps to determine the pattern of GM atrophy in MCI patients. The statistical threshold was set at *p* < 0.05 using the AlphaSim correction for multiple comparisons with a threshold of *p* < 0.01 at voxel level and a minimum cluster size of 1492 voxels.

### Between-Group Comparison of ReHo in Resting and Working Memory States

To determine the interaction effects of group and cognitive state on ReHo, we performed a 2-way repeated-measures analysis of variance (ANOVA) on a voxel-by-voxel basis using SPM8, with group (MCI and NC) as a between-subject factor and cognitive state (resting and working memory) as a repeated-measure, controlling for age, gender, education, and head motion. *Post hoc* 2-sample *t*-tests were performed on clusters showing significant group × state interactions. The statistical threshold was set at *p* < 0.05 using the AlphaSim correction for multiple comparisons with a threshold of *p* < 0.01 at the voxel level and a minimum cluster size of 85 voxels (recommended by DPABI). All coordinates are reported in the MNI format.

### Correlation of State-Related Changes with Working Memory Performance

We calculated Pearson correlation coefficients (*p* < 0.05) to explore the relationship between state-related changes in ReHo and working memory performance (response accuracy divided by RT) in the MCI group in regions that showed a significant interaction between group and state. Bootstrap results were based on 1000 bootstrap samples, and 95% confidence intervals are reported.

### Functional Connectivity (FC) Analysis

To examine changes in FC between resting and task states in regions showing significant group × state interactions, we conducted a seed-based connectivity analysis with regions showing group × state interactions in all frequency bands as seeds. For both resting and task states in each MCI or NC individual, voxel-wise FC maps to a given seed were computed as maps of temporal correlation coefficients between the BOLD time course of each voxel and the averaged BOLD time course across voxels in the seed region. FC maps from individual subjects were then transformed using Fisher’s *Z* transformation for group-level *t*-tests.

## Results

### Frequency Bands Slow-2 and Slow-4

We did not observe any stable regions with a significant interaction between group and state in the slow-2 or slow-4 frequency bands.

### Frequency Band Slow-3

**Figure 1:**
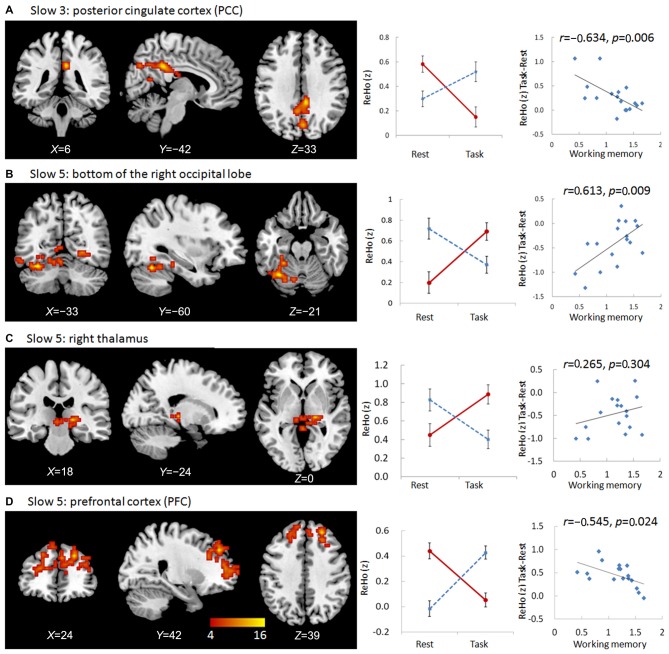
**Regions showing group × state interactions for ReHo including the posterior cingulate cortex (PCC) in slow-3 (A), and the bottom of the right occipital lobe (B), the right thalamus (C), and the prefrontal cortex (PFC; D) in slow-5.** The color bar demonstrates *F* values. The corresponding graphs show the interaction patterns (middle panels) and correlation (right panels) between state-related change (task minus resting) of ReHo and working memory performance in mild cognitive impairment (MCI) patients.

We observed significant interactions between group and state in the bilateral and especially right posterior cingulate cortex (PCC) in the slow-3 frequency band (Figure 1, Table 2). Further *post hoc* *t*-tests revealed that the value of ReHo was decreased in the working memory state relative to the resting state in NCs, but increased in MCI patients. Furthermore, the state-related change (task minus resting) of ReHo in this cluster correlated negatively with working memory performance in MCI patients (*r* = −0.634, *p* = 0.006, bootstrap-based 95% confidence interval −0.873, −0.241; Figure 1). These data indicated that MCI patients with greater working memory impairment showed a stronger decrease in ReHo between the resting and task states. Correlations were not significant in the NC group (*p* > 0.10 for all comparisons).

**Table 2:** **Regions showing group (MCI, NC) × state (resting, working memory task) interaction for ReHo in slow-3 and slow-5 bands**.

Regions	Peak (MNI coordinates)			*F*-value	Cluster size
	*x*	*y*	*z*
Slow-3
Right PCC	6	−42	33	14.60	96
Slow-5
Bottom of the right medial occipital lobe and part of the right cerebellum	−33	−60	−21	18.68	450
Right thalamus	18	−24	0	18.14	109
Bilateral PFC	24	42	39	24.10	840
Left precuneus	−24	−72	48	14.37	141
Right precuneus	24	−93	33	11.81	98
Right angular gyrus	39	−60	42	14.85	90

*Abbreviations: PPC, posterior cingulate cortex; PFC, prefrontal cortex; MNI, Montreal Neurological Institute*.

### Frequency Band Slow-5

**Figure 2:**
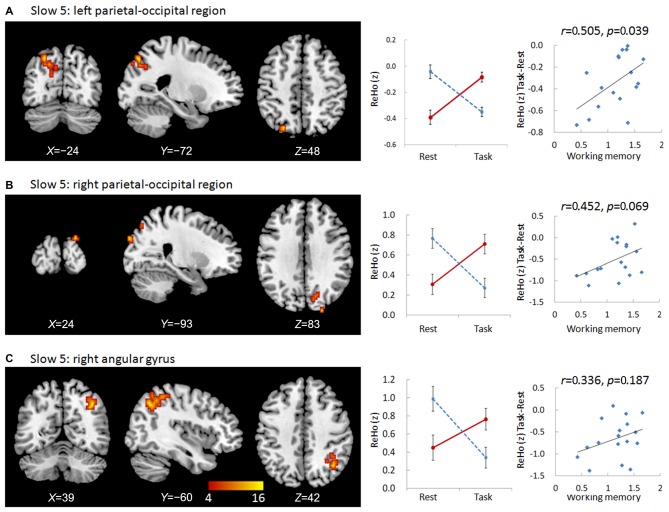
**Regions showing group × state interactions for ReHo in slow-5 including the left parietal-occipital region (A), the right parietal-occipital region (B), and the right angular gyrus (C).** The color bar demonstrates *F* values. The corresponding graphs show the interaction patterns (middle panels) and correlation (right panels) between state-related change (task minus resting) of ReHo and working memory performance in MCI patients.

We observed significant interactions between group and state in six regions in the slow-5 frequency band: the bottom of the right occipital lobe, the right thalamus, a diffusing region in the bilateral prefrontal cortex (PFC), the left and right parietal-occipital regions, and the right angular gyrus (Figures 1, 2). Further *post hoc* *t*-tests revealed that, in the PFC, the value of ReHo was decreased in the working memory state relative to the resting state in NCs, but increased in MCI patients. In contrast, this pattern was reversed in the other five clusters. That is, the value of ReHo was increased in the working memory state relative to the resting state in NCs, but decreased in MCI patients (Figures 1, 2). Furthermore, correlations between state-related changes of ReHo and working memory performance were significant in the bottom of the right occipital lobe (*r* = 0.613, *p* = 0.009, bootstrap-based 95% confidence interval 0.212, 0.847), the PFC (*r* = −0.545, *p* = 0.024, bootstrap-based 95% confidence interval −0.0889, −0.144), the left parietal-occipital region (*r* = 0.505, *p* = 0.039, bootstrap-based 95% confidence interval 0.071, 0.819), and the right parietal-occipital region (marginally, *r* = 0.452, *p* = 0.069, bootstrap-based 95% confidence interval 0.014, 0.761). Correlations did not reach significance in the right thalamus (*r* = 0.265, *p* = 0.304, bootstrap-based 95% confidence interval −0.439, 0.729) or the right angular gyrus (*r* = 0.336, *p* = 0.187, bootstrap-based 95% confidence interval −0.174, 0.759; Figures 1, 2). Correlations were not significant in any clusters in the NC group (*p* > 0.100 for all comparisons).

### VBM Analysis

**Figure 3:**
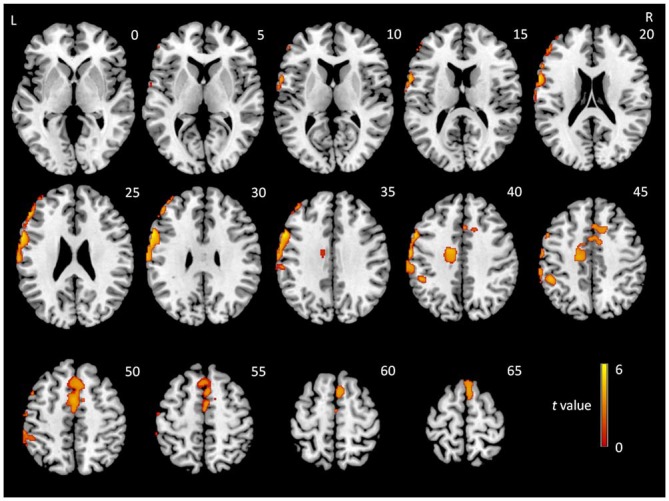
***t*-Statistical difference maps in gray matter (GM) volume between MCI patients and normal controls (NCs).** Results were obtained using a two-sample *t*-test. The color bar demonstrates *t* values.

To examine whether the regions showing interactions between group and state were affected by GM atrophy in MCI patients, a VBM analysis was conducted. MCI patients exhibited significant GM loss in two brain regions relative to NCs: the middle part of the medial frontal lobe, including parts of the cingulate gyrus, the limbic lobe, and nearby white matter (peak MNI coordinates: *x* = −58.5, *y* = 12, *z* = 33; *t* = 4.12; cluster size = 2447) and the lateral frontal and parietal lobes (peak MNI coordinates: *x* = −18, *y* = −7.5, *z* = 42; *t* = 3.14; cluster size = 2177; Figure 3).

Regions with GM atrophy in MCI patients did not appear to overlap with regions showing significant interactions between group and state. To confirm this observation, two-sample *t*-tests (*p* < 0.05) were conducted to compare the GM atrophy in MCI patients in all regions showing group × state interactions in each frequency band. The AlphaSim correction was used for multiple comparisons with a threshold of *p* < 0.01 at the voxel level and a minimum cluster size was estimated using the DPABI. None of the results reached significance.

To further confirm interaction effects and to control for GM atrophy in MCI patients, we extracted the time course of each region showing a group × state interaction in each frequency band, and conducted an additional ANOVA with age, gender, education, head motion, and GM intensity as covariates. The results confirmed our findings described above.

### Functional Connectivity of Regions Showing Group × State Interactions

**Figure 4:**
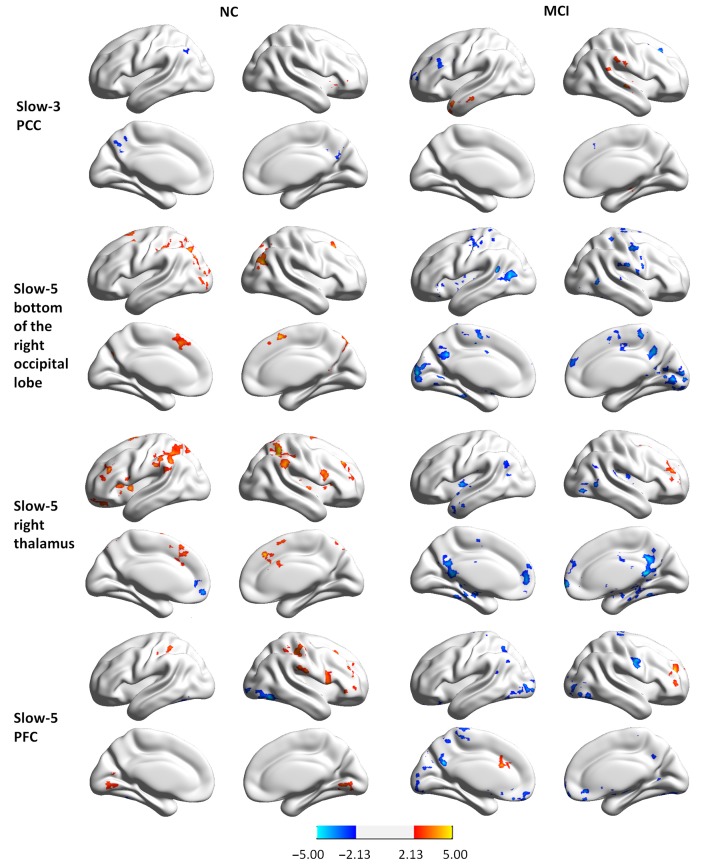
**Group differences in seed-based connectivity maps in resting and task states for four clusters showing group × state interactions: the PCC in slow-3, and the bottom of the right occipital lobe, the right thalamus, and the PFC in slow-5**.

**Figure 5:**
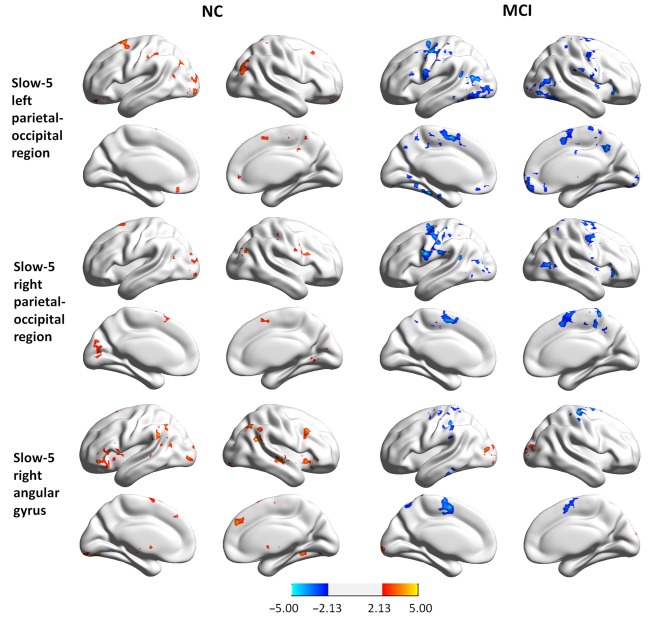
**Group differences in seed-based connectivity maps in resting and task states for three clusters showing group × state interactions: the left parietal-occipital regions, the right parietal-occipital regions, and the right angular gyrus in slow-5**.

To examine whether abnormal ReHo in regions showing significant interactions between group and state was partially the result of deficits in distant FC, we conducted a seed-based connectivity analysis with regions showing group × state interactions as seeds. Seed-based connectivity maps (*p* < 0.01 and cluster size > 85 voxels) for each region are shown in the Supplementary Materials (Figures S1–S3). It should be noted that none of the negative connectivities reached significance according to our conditions. Thus, only the positive connectivities are presented. Difference maps comparing groups in each state (paired *t*-test *p* < 0.05, 85 voxels) are shown in Figures 4, 5.

MCI patients demonstrated altered FC patterns as compared to NCs. In NC subjects, the connectivity maps at slow-3 (the PCC) and slow-5 (the bottom of the right occipital lobe, the PFC, the left and right parietal-occipital regions, and the right angular gyrus) were more diffuse during tasks relative to the resting state. In contrast, the connectivity maps of MCI patients were more focal during tasks relative to the resting state. Furthermore, difference maps comparing the state for each group showed a consistent pattern in slow-5: in NC subjects, most of the clusters demonstrated increased FC during tasks relative to the resting state (red regions in Figures 4, 5), while clusters demonstrated decreased FC in MCI patients (blue regions in Figures 4, 5).

## Discussion

The present study demonstrates frequency-dependent alterations in ReHo in NC subjects and MCI patients during resting and working memory states. No brain regions demonstrated significant interactions between group and state in the slow-2 and slow-4 frequency bands, but significant interactions were observed in the PCC in the slow-3 frequency band, and in six regions in the slow-5 frequency band (the bottom of the right occipital lobe and part of the right cerebellum, the right thalamus, a diffusing region in the bilateral PFC, the left and right parietal-occipital regions, and the right angular gyrus). Furthermore, patterns of interaction between group and state in these regions were dependent upon frequency band. Specifically, the value of ReHo was decreased in the working memory state in NCs but increased in MCI patients in the PCC in slow-3 and in the PFC in slow-5. In contrast, the value of ReHo was generally increased in the working memory state in NCs but decreased in MCI patients in slow-5. That is, alterations in ReHo in the working memory state were distinct in different frequency bands of brain oscillation.

It should be noted that our study accounted for the possible influence of GM structural atrophy on our findings. A VBM analysis indicated that while MCI patients indeed demonstrated GM atrophy in the middle part of the medial frontal lobe and in the lateral parts of frontal and parietal lobes, these regions did not overlap with regions showing interactions between group and state. Our observations of GM atrophy are similar to the results of previous studies in MCI patients (Singh et al., 2006; Karas et al., 2008). While studies have indicated that atrophy of the structural fiber tracts accompanies the breakdown of activation patterns during working memory performance in MCI patients (Teipel et al., 2015), ReHo alterations in the present study are speculated to be related to deficits in FC (see below) rather than structural atrophy.

In our study, state-related changes in ReHo correlated with working memory performance in MCI patients in all but two clusters (the right thalamus and the right angular gyrus in slow-5). Importantly, the performance-associated pattern observed in MCI patients always demonstrated an opposing trend in NC subjects. This indicates that the altered pattern of ReHo in MCI patients during the working memory state was not a compensatory mechanism for impaired cognitive function, but rather directly related to impairment. Of note, none of the correlations between state-related changes in ReHo and working memory performance reached significance in the NC group. This may due to a focal distribution of data in MCI patients vs. a diffuse distribution in NC subjects.

As a key structure in working memory processing, the PFC exerts control over behavior by biasing the salience of mnemonic representations and adjudicating among competing context-dependent rules (D’Esposito and Postle, 2015). Previous studies found that MCI patients show significantly decreased activity in the PFC and significantly increased activity in the parietal-occipital regions during working memory tasks (Bokde et al., 2010; Lou et al., 2015). Consistent with these findings, the PFC and parietal occipital-regions showed group × state interactions in our study. MCI patients indeed show decreased activity in regions around the middle occipital gyrus and the middle frontal gyrus (reported in another article Yu et al., under review). In addition, the PCC region in slow-3 has been demonstrated to be a part of the default mode network (DMN; Greicius et al., 2003), and the diffusing clusters in the PFC in slow-5 were also included in the DMN, whereas the angular gyrus and the bilateral parietal-occipital regions have been reported to be parts of the ventral attention network (Margulies and Petrides, 2013; Bartolomeo, 2014; Patel et al., 2015). The results of the present study therefore indicate that MCI patients demonstrate abnormal intra-regional synchronization in the DMN in a relatively high-frequency band, and abnormal intra-regional synchronization in both the DMN and the attentional network in a relatively low-frequency band. It is interesting that, in NC subjects, the value of ReHo in DMN clusters decreased while the value of ReHo in clusters belonging to the attentional network increased independent of frequency during tasks. This finding is consistent with the notion that the DMN is active in the resting state but inhibited during task processing. The reversal of this pattern in MCI patients (i.e., hyper-activity of the DMN and hypo-activity of the attentional network during tasks) was associated with working memory performance, indicating that working memory impairments in MCI are not only related to abnormal activity responses to external stimuli, but also abnormal oscillations of brain networks during the task state.

Given the observation of abnormal intra-regional synchronization in MCI patients, we considered whether inter-regional synchronization was also impaired in regions showing group × state interactions. The results demonstrated that FC was indeed altered in these regions compared with other parts of the whole brain in MCI patients, although the pattern of alterations changed and even reversed according to the location of the clusters and the frequency band. Generally, connectivity maps were more diffuse during the task relative to the resting state for NC. In contrast, the connectivity maps of MCI patients were more focal during tasks relative to resting state. Furthermore, difference maps comparing the resting vs. task states showed increased FC during the tasks in NC subjects but decreased FC in MCI patients. These data indicate that specific networks became more segregated when MCI patients were in a cognitive state constrained by working memory.

Brain oscillations occur over a wide range of frequencies. It has been suggested that each oscillatory band is generated by different mechanisms and relates to different physiological functions (Buzsáki and Draguhn, 2004; Zuo et al., 2010). Several studies have observed alterations of the brain network in MCI patients during the resting and task states in relatively high-frequency bands using EEG (Pijnenburg et al., 2004; Deiber et al., 2009) and magnetoencephalography (López et al., 2014). Additionally, an fMRI study reported brain network alterations in relatively low frequency bands in MCI patients during the resting state (Han et al., 2011). These previous studies suggested the potential use of the slow-5 band for disease diagnosis and progression monitoring in MCI patients. Alternatively, the present study demonstrates brain network alterations in relatively low-frequency bands in MCI patients during the working memory state. Our work therefore expands previous work by adding another factor, i.e., state-related change, which may also serve as a parameter for disease diagnosis and progression monitoring in MCI patients. Further studies are needed to explore other frequency-dependent and frequency-independent characteristics of brain networks (such as ALFF and degree centrality) in MCI patients during the working memory state.

The limitations of the present meta-analysis are largely related to the small number of MCI patients. The subtypes and the severity of cognitive impairment in MCI patients could have a significant impact on patterns of brain oscillation, and this issue should be considered in future studies. In addition, the working memory component of our task was not very pure because processing consisted of encoding, retrieval, and sub-resting sessions (for details, see “Materials and Methods” Section). Given the observation of decreased activity in different regions during the encoding and retrieval processes of a spatial working memory task in MCI patients (Lou et al., 2015), future studies should separate these two subprocesses to explore whether brain oscillations vary according to these subtypes in working memory states.

To our knowledge, this is the first study to examine alterations in ReHo in MCI patients and identify frequency-dependent effects during working memory states. The results provide a new perspective regarding the neural mechanisms of working memory deficits in MCI patients, and extend our knowledge of altered brain patterns in resting and task-evoked states.

## Author Contributions

PW conceived the idea, designed the study, analyzed and interpreted data, and drafted part of the manuscript. RL and ZH assisted the analysis and interpretation of data. JY conducted the experiment and drafted part of the manuscript. JL conceived the idea, designed the study, and participated in the writing and revision of the manuscript.

## Funding

This research was supported by the National Natural Science Foundation of China (31271108, 30911120494, 31070916 and 31400895); the National Science and Technology Pillar Program of China (2009BAI77B03); the Knowledge Innovation Project of the Chinese Academy of Sciences (KSCX2-EW-J-8), CAS/SAFEA International Partnership Program for Creative Research Team (Y2CX131003), the Institute of psychology, Chinese Academy of Sciences (111000C038, Y3CX151005), and Key Laboratory of Mental Health, Institute of Psychology, Chinese Academy of Sciences (KLMH2014ZK02, KLMH2014ZG10).

## Conflict of Interest Statement

The authors declare that the research was conducted in the absence of any commercial or financial relationships that could be construed as a potential conflict of interest.

## Supplementary Material

The Supplementary Material for this article can be found online at: http://journal.frontiersin.org/article/10.3389/fnagi.2016.00060/abstract

## Footnotes

^1^http://www.fil.ion.ucl.ac.uk/spm

^2^http://rfmri.org/dpabi
