# Supplementary material for: Frequency-Dependent Brain Regional Homogeneity Alterations in Patients with Mild Cognitive Impairment during Working Memory State Relative to Resting State
Source: Front Aging Neurosci. 2016 Mar 24;8:60. doi: 10.3389/fnagi.2016.00060 (PMC4805610; doi:10.3389/fnagi.2016.00060)
Supplement: Supplementary file 1 [file Image_1.pdf]

Supplementary Material

Frequency-dependent brain regional homogeneity alteration in mild cognitive impairment during working memory state relative to resting state

Pengyun Wang [1], Rui Li [1], Jing Yu [2], Zirui Huang [3], and Juan Li [1] *

[1] Center on Aging Psychology, Key Laboratory of Mental Health, Institute of Psychology, Chinese Academy of Sciences, Beijing, 100101, China

[2] Xinan University, China

[3] Institute of Mental Health Research, University of Ottawa, Ottawa, Canada

*****Corresponding** to lijuan@psych.ac.cn

Supplementary Figures

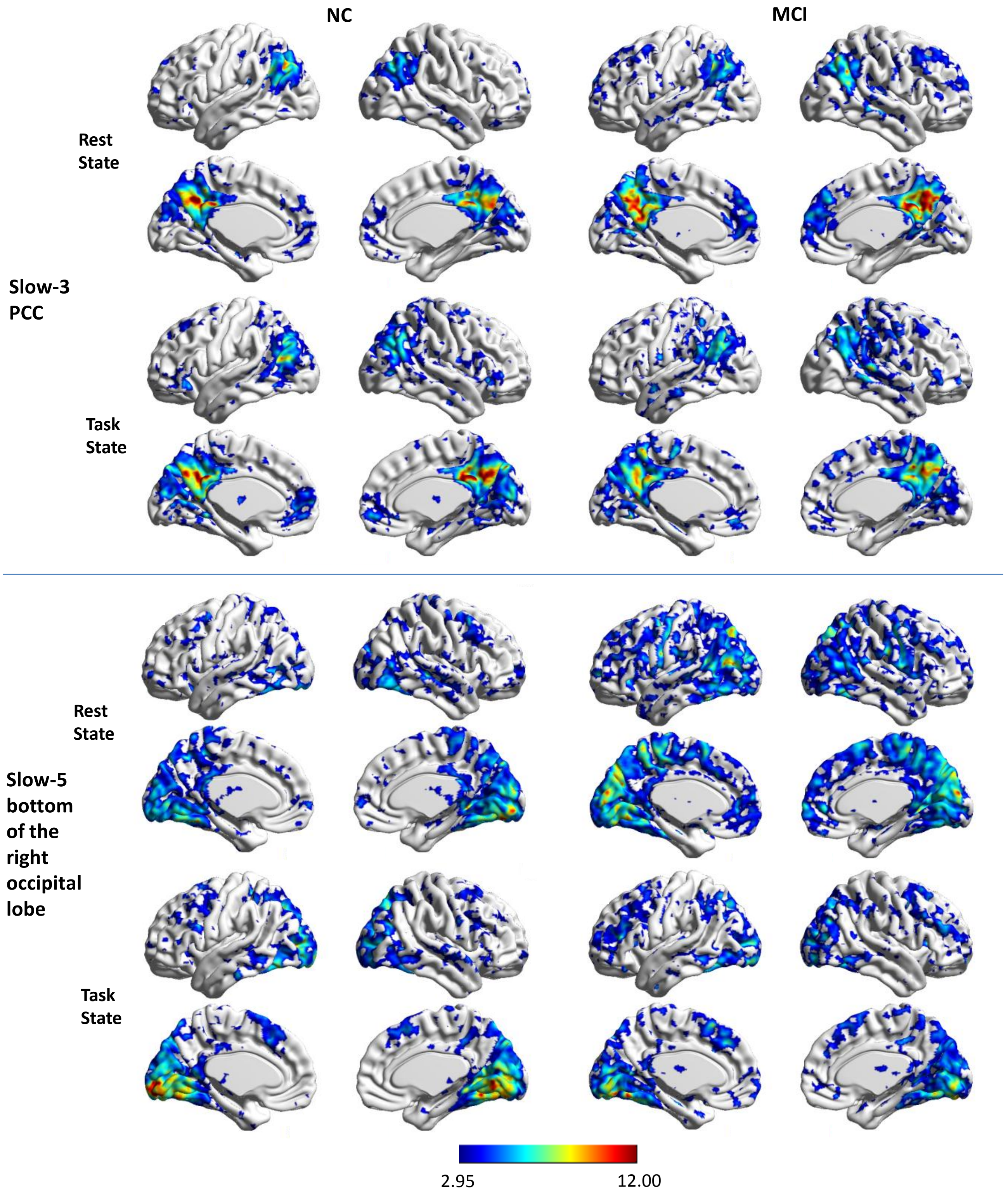

Supplemental sFig. 1. Seed-based connectivity maps of distant functional connectivity patterns in resting and task states in NC and MCI patients for two clusters showing group × state interactions: posterior cingulate cortex (PCC) in slow-3, and the bottom of the right occipital lobe in slow-5.

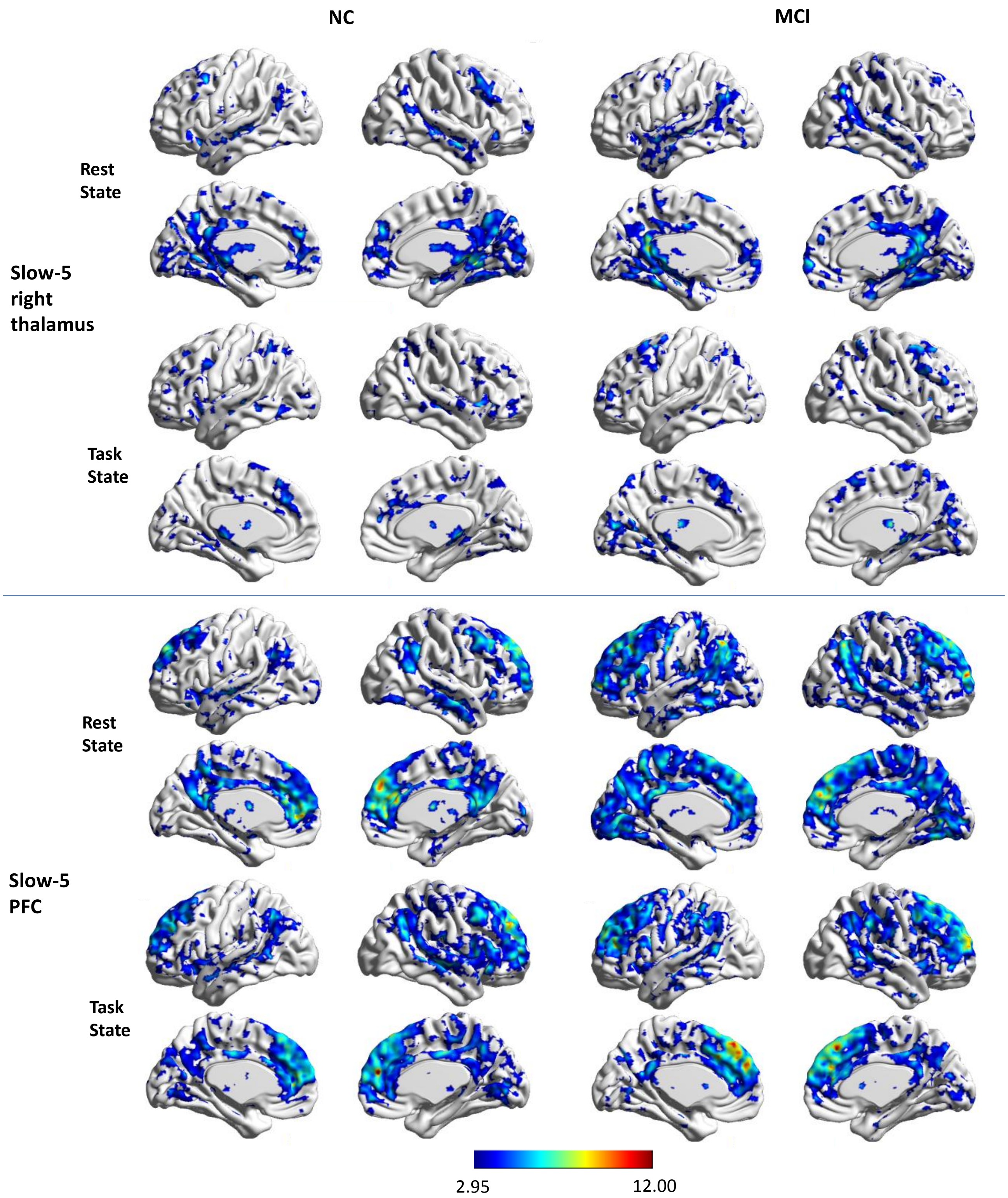

Supplemental sFig. 2. Seed-based connectivity maps of distant functional connectivity patterns in resting and task states in NC and MCI patients for two clusters showing group × state interactions: right thalamus, prefrontal cortex (PFC) in slow-5.

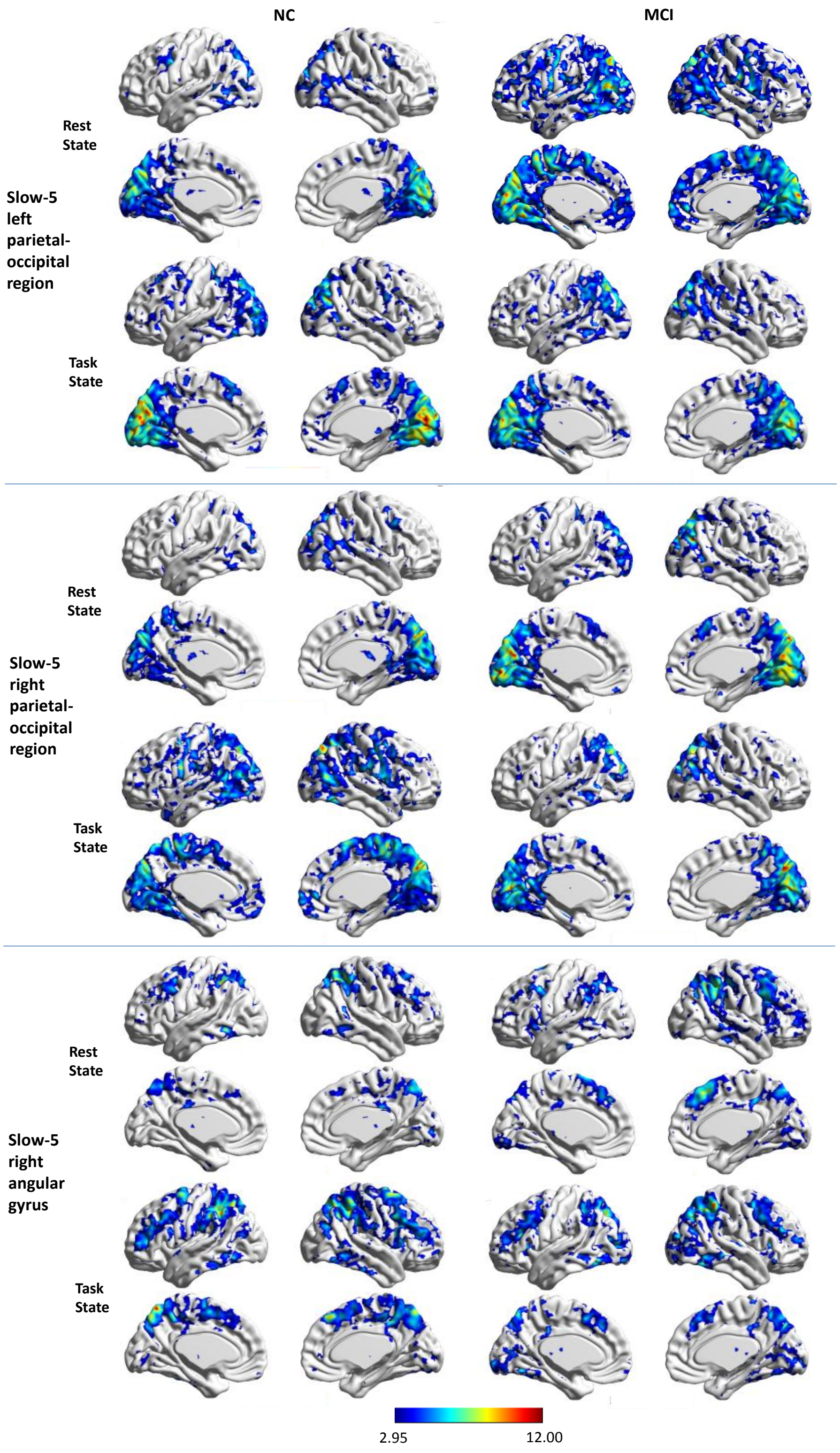

Supplemental sFig. 3. Seed-based connectivity maps of distant functional connectivity patterns in resting and task states in NC and MCI patients for three clusters showing group × state interactions: left parietal-occipital regions, right parietal-occipital regions, and right angular gyrus in slow-5.
